# Construction of AC/DC magnetic syringe device for stimulated drug release, injection and ejection of nanocarriers and testing cytotoxicity *in vitro*

**DOI:** 10.1016/j.mex.2021.101312

**Published:** 2021-03-21

**Authors:** Milorad Zjalić, Mislav Mustapić, Zvonko Glumac, Ivan Prološčić, Senka Blažetić, Ana Vuković, Mostafa Masud, Motasim Billah, Aslam Khan, Suzana Šegota, Md Shahriar Al Hossain, Marija Heffer

**Affiliations:** a Department of Medical Biology and Genetics, Faculty of Medicine, J.J. Strossmayer University of Osijek, J. Huttlera 4, 31 000 Osijek, Croatia; b Department of Physics, J.J. Strossmayer University of Osijek, Trg Ljudevita Gaja 6, Osijek 31000, Croatia; c Department of Biology, J.J. Strossmayer University of Osijek, Ulica cara Hadrijana 8a, Osijek 31000, Croatia; d Australian Institute for Bioengineering and Nanotechnology, University of Queensland, St. Lucia, Brisbane QLD 4067, Australia; e School of Mechanical and Mining Engineering, University of Queensland, St. Lucia, Brisbane QLD 4067, Australia; f King Abdullah Institute for Nanotechnology, King Saud University, Riyadh 11451, Saudi Arabia; g Division of Physical Chemistry, Ruđer Bošković Institute, Zagreb 10000, Croatia

**Keywords:** Metal nanoparticles, Drug delivery systems, Cell viability, Oxidative stress, Lipid peroxidation

## Abstract

Iron nanoparticles are used as a targeted drug delivery system. The nanocarrier itself can be genotoxic, trigger oxidative stress or cell death. Therefore, we developed an AC/DC magnetic syringe for injecting, stimulating drug release and safe removing of the nanocarrier. Alongside we optimized the method for nanoparticles’ drug release kinetics and testing cytotoxicity in vitro.

• This paper presents detailed instructions for construction of AC/DC magnetic syringe device for stimulated drug release, injection and ejection of magnetic nanoparticles; nanoparticles preparation; adsorbing methylene blue on nanoparticles; determination of drug release kinetics from nanocarriers on the example of methylene blue
• Gomori´s Prussian blue reaction for differentiated SH-SY5Y human neuroblastoma cell line; MTT viability assay optimized for differentiated SH-SY5Y human neuroblastoma cell line and antioxidant enzymes activities assay and lipid peroxidation methods are optimized for cell analyses cell cultivation for nanoparticles cytotoxicity testing *in vitro*.
• Those protocols are the first step toward further testing the effect of nanoparticles *in vivo*, on brain tissue.

## Graphical abstract

**Figure:**
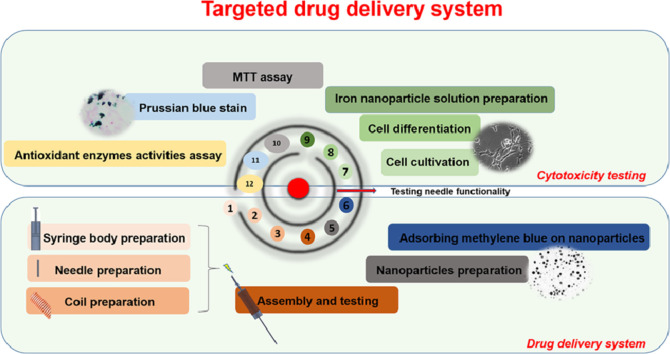

Specifications table

**Table:** 

Subject Area:	Medicine and Dentistry Medicine and Dentistry
More specific subject area:	*Targeted drug delivery*
Method name:	*1. Construction of AC/DC magnetic syringe device for stimulated drug release, injection and ejection of magnetic nanoparticles.* *2. Nanoparticles preparation.* *3. Adsorbing methylene blue on nanoparticles.* *4. Determination of drug release kinetics from nanocarriers on the example of methylene blue.* *5. Cell cultivation for nanoparticles cytotoxicity testing in vitro.* *6. Gomori´s Prussian blue reaction optimized for differentiated SH-SY5Y human neuroblastoma cell line.* *7. MTT viability assay optimized for differentiated SH-SY5Y human neuroblastoma cell line.* *8. Antioxidant enzymes activities assay and lipid peroxidation optimized for cell culture.*
Name and reference of original method:	*[Mustapić et al., 2016] Mustapić, M., Hossain, Md. Sh., Horvat, J., Wagner, P., Mitchell, D.R.G, Kim, J.H., Alici, G., Nakayama, Y., Martinac, B., 2016. Controlled delivery of drugs adsorbed onto porous Fe3O4 structures by application of AC/DC magnetic fields, Micropor. Mesopor. Mat., 226, 243–250. 10.1016/j.micromeso.2015.12.032.* *[Šegota et al., 2019] The role of spin-phonon coupling in enhanced desorption kinetics of antioxidant flavonols from magnetic nanoparticles aggregates Šegota S, Baranović G, Mustapić M, Strasser V, Domazet Jurašin D, Crnolatac I, Hossain Md Sh, Dutour Sikirića M, Journal of Magnetism and Magnetic Materials 490 (2019) 165530.* *[Vacca, 1985] Vacca, L., 1985. Gomori's Prussian blue reaction, in Vacca, L. Laboratory manual of histochemistry. Raven Press, New York, pp.396–397. ISBN: 0890055408, 9780890055403.* *Abcam, MTT assay protocol.*https://www.abcam.com/kits/mtt-assay-protocol/*[Dolphin et al., 1989]* Dolphin, D., Poulson, R., Avramović, O., Glutathione: Chemical, biochemical, and medical aspects. John Wiley & Sons Inc., New York. 1989.*cessed 9 October 2020).* *[Ohkawa et al., 1979]* Ohkawa, H., Ohishi, N., Yagi. K., Assay for lipid peroxides in animal tissues by the thiobarbituric acid reaction. Anal Biochem. 95, (1979) 351–8. https://doi:10.1016/0003-2697(79)90738–3.
Resource availability:	

## Method details

**Construction of AC/DC magnetic syringe device for stimulated drug release, injection and ejection of magnetic nanoparticles**

### Material

Note: Materials are listed in order that they appear in procedure steps.

• Permalloy, supermalloy or soft iron rod 150 mm long, 5–8 mm in a diameter
• Lathe machine
• Medical grade PTFE rod 100 mm long, 6 mm in a diameter
• Needle with Luer type metal hub made from permalloy; G26, 35 mm in length
• 5 meters of lacquer insulated 23 AWG copper wire
• Copper wire, AWG 20, silicone insulation coated – 2 m
• Kapton tape 1 cm wide – 5 m roll
• Safety-type banana jacks – male – 2 pieces
• Thermal shrink insulating sleeves, 3 mm in diameter – 10 pcs
• Signal generator with variable voltage, current and frequency (min. req. 0–30 V, 0.1-2A, 1 Hz–50 kHz)
• Fast curing epoxy resin
• Laboratory oven

### Procedure

#### Magnetic syringe body preparation

The syringe should be made preferably from permalloy to allow maximal induction and transfer of electromagnetic fields to the needle. The composition of permalloy varies but it can be described as an alloy containing 80% of nickel and 20% of iron what makes it highly magnetic [1,2]. Other materials suitable for this are supermalloy or soft iron. Since the majority of commercially available medical steel used in syringe production is steel 304 or steel 316, both austenitic and paramagnetic, the syringe has to be specially made [3,4]. Syringe body should be produced by turning 150  mm long, 5–8 mm in diameter wide permalloy rod on a lathe machine. The syringe length should be 100 mm, with an inner diameter of 3.25  mm and an outer diameter of 6.00  mm. The tip of the syringe must conform to the Luer tip (LT) specifications. Tip outer diameter can range from 3.924 – 4.026 mm according to ISO 594-1 Luer standard. Tip inner diameter should be 2  mm. The piston should be turned on a lathe machine out of medical-grade PTFE with a final diameter of 3.24  mm. PTFE is a good material of choice because it is inert to most chemicals can and it has a hydrophobic property which makes it very good in creating seal if crafted to tight dimensional specifications [5]. Acceptable tolerances for both syringe body and piston should not exceed ±0.005  mm. The syringe and the needle are to be made from permalloy. This design is intended for use in ‘*in vitro’* systems. Additional measures must be taken for use in ‘*in vivo’* systems. For example, the inner chamber of the syringe could be immerse coated in a thin layer of medical-grade silicone to prevent an allergic reaction due to the high nickel concentration in permalloy [6].

#### Needle preparation

The needle has to be specially ordered from manufacturers. It must have Luer type metal hub made from permalloy to properly fit onto the syringe. The needle length is 35 mm and the thickness of the needle is G26. Before use, the needle should be sterilized at 121 °C for 20 min.

#### Coil preparation

Coil dimensions inner diameter is 6 mm, 6.5 cm in length made out of 1000 turns in 9 layers, 112 winds per layer [7,8].

1. To make the coil, use 23 AWG varnish insulated copper wire. The necessary length is around 4 m.
2. On the syringe body measure 6.5 cm length for the coil and mark the length on it.
3. Measure 15 cm of wire and bend it 90°.
4. Place bent wire at one of the marked ends. (Note: Shorter part of wire must point outwards since it will become coil terminal.)
5. Start coiling the longer part of the wire tightly without spacing between turns.
6. When the first layer is finished, use a piece of Kapton tape to temporary fix the wire in position before start winding the second layer.
7. In the end, measure 15 cm of wire for the terminal and fix it temporarily.
8. Cover the entire coil with 2 layers of Kapton tape.
9. Slide the prepared coil from a mould and set it aside.
10. Once everything is prepared, connect the coil terminals to (silicone insulated) wire by soldering. 2 cm of terminals of coil wire must be burned off to remove the varnish and to allow proper soldering and securely isolate naked wires with shrink-wrap isolation sleeves. The wire must have on the other end safety-type banana jacks.

#### Assembly and testing


 

1. Slide the coil onto the syringe body.
2. Fit the needle onto the syringe.
3. Connect everything into the laboratory power supply with variable voltage, current and frequency.
4. Let current through the coil and test magnetization of the needle.
5. Move the coil up and down the syringe body until the best inductance is achieved.
6. Fix the coil in the place with fast curing epoxy resin. Apply resin in a dot-like manner. 2 dots per each side of the coil are sufficient.
7. After use, a syringe with a coil on it should be sterilized in an autoclave at 105 °C for a 20-min duration and dried in a laboratory oven at 50 °C for the 2-h duration.

## Nanoparticles preparation

### Material

Note: Materials are listed in the order that they appear in procedure steps.

• Hexadecyltrimethylammonium bromide (CTAB) (Sigma-Aldrich, St. Louis, MO, SAD)
• Sodium dodecyl sulphate (SDS) (Sigma-Aldrich, St. Louis, MO, SAD) CAT# 219374-100GM
• Distillate water
• Cyclohexane (Sigma-Aldrich, St. Louis, MO, SAD) CAT# 436143-100G
• Glass laboratory beaker 100 mL volume
• Magnetic stirrer
• Gas tanks with nitrogen
• Gas purging equipment: round bottom flask, rubber stopper, 2 metal or glass 4mm wide tubes
• Iron (II)chloride (Sigma-Aldrich, St. Louis, MO, SAD) CAT# 450936-25G
• Gas tanks with argon or helium
• Iron (III)chloride (Sigma-Aldrich, St. Louis, MO, SAD) CAT# 451649-5G
• 30% ammonia solution (Sigma-Aldrich, St. Louis, MO, SAD) CAS Number: 1336-21-6
• Inert atmosphere glovebox
• Vortex
• Magnetic stick
• Acetone
• Laboratory oven

### Procedure

The preparation of nanoparticles is described by Mustapić et. All in a previous publication [7]. A slight deviation from the synthesis protocol is to be made to achieve the highest gains.

1. Prepare a detergent solution by mixing 1.5 g of the cationic surfactant hexadecyltrimethylammonium bromide (CTAB) and 1.5 g of the anionic surfactant sodium dodecyl sulphate (SDS) with 30 ml of water.
2. In detergent solution add 60 ml of cyclohexane place on a magnetic stirrer until detergents are dissolved remove from stirrer and precede with degassing in N_2_ atmosphere by gently blowing a nitrogen gas through the solution.
3. Gas purging equipment is assembled by drilling two holes in a rubber stopper and pushing two metal or glass 4 mm wide tubes through the holes and placing everything on a round bottom flask. One tube is longer and should be immersed in the solution, the second tube is short and bent 180° on the outer end.
4. Gas is blown through a long tube in the solution and released through short out from the flask.
5. During the period of degassing of surfactants prepare 20 ml of 0.0015 mol/L of iron (II) chloride solution in type 1 water under argon or another inert gas atmosphere. (Note: The inert gas atmosphere is to prevent further oxidation of iron (II) chloride).
6. Prepare 20 ml of 0.003 mol/L of iron (III) chloride solution in type 1 water. (Note: The inert gas atmosphere is not required for iron (III) chloride because it is in the highest oxidation state already.)
7. Stir both solutions on a magnetic stirrer for half an hour.
8. In an inert gas atmosphere in a separate flask mix prepared iron chloride solutions and start adding into it 5 ml of 30% ammonia and do so for 1 min.
9. After 1 min of a reaction pour the detergent solution in the flask and gently mix by hand vortexing.
10. After 5 min reaction has finished and formed nanoparticles can be collected from the bottom of the flask by using an external permanent magnet.
11. Wash NP several times – alternatively first with type 1 water and then with an acetone, the washing procedure must end with an acetone. (Note: in this step centrifuge can be used, minimum 5 min at 10 000 g).
12. Dry NP overnight in a laboratory oven at 60 °C.
13. Collect black powder and place it in a laboratory oven at 250 °C in 2-h duration to remove detergent under an inert atmosphere.

## Adsorbing methylene blue on nanoparticles

### Material

Note: Materials are listed in the order that they appear in procedure steps.

• Methylene blue (Carl Roth, Karlsruhe, Germany) Art. No. A514.1
• Milli-Q^Ⓡ^ or other Type 1 ultrapure water
• Flask 100 ml with ground glass neck and corresponding cap
• 100 mg of Fe_3_O_4_ nanoparticles
• Duran bottle caps GL45 (Duran, Duran, Birmingham, UK) – 9 pieces Silica gel Gas/vacuum chamber
• Nitrogen or other inert gas
• Laboratory paraffin film Parafilm^Ⓡ^ (Sigma-Aldrich, St. Louis, MO, SAD)
• Rare earth magnet
• Freeze dry dehydrator (Note: If freeze dry apparatus is not available, you will need: 50 ml Falcon tube place, 50 g of anhydrous calcium sulphate or silica gel, cotton wool 1 cm thick and Eppendorf tube)

### Procedure

In this protocol, we have chosen to adsorb methylene blue on the nanoparticles, but any active and easily measurable substance can be used. When using other substances expect protocol adjustment to be made.

1. Prepare 20 ml 0.001M aqueous solution of methylene blue (MB) in type 1 grade water.
2. Place it in a flask with a ground-glass neck.
3. Weight 100 mg of Fe_3_O_4_ nanoparticles (NP).
4. Place NP in MB solution.
5. De-gas solution under medium vacuum (1 × 10^3^ Pa) in 1-h duration at +4 °C.
6. During pressure, release nitrogen or other inert gas to devoid solution out of residual oxygen. (Note: It serves to prevent additional oxidations due to prolonged exposure to water.)
7. Take out the flask and cap it with a corresponding ground glass cap.
8. Wrap cap and neck of the flask with laboratory paraffin film.
9. Leave NP in degassed MB solution overnight to saturate at room temperature.
10. The next day gathers nanoparticles on one spot by using a rare earth magnet.
11. Wash NP 4 times with type 1 grade water for 10 min each, or until water runs clear. Use at least 50 ml of water per wash. (Note: This step serves to remove any loosely bound MB.)
12. Remove water and freeze-dry prepared nanoparticles. (Note: If freeze dry apparatus is not available, at the bottom of 50 ml Falcon tube place 50 g of anhydrous calcium sulphate or silica gel. On top of it place a wad of cotton wool 1 cm thick. Place open Eppendorf tube into the cotton wool and fix it firmly. Close the Falcon tube and place everything in -80 °C freezer for 7 days to completely dry NP) [7].

## Determination of drug release kinetics from nanocarriers on the example of methylene blue

### Material

Note: Materials are listed in the order that they appear in procedure steps.

• Glass beaker 50 ml
• Milli-Q^Ⓡ^ or other Type 1 ultrapure water
• Methylene blue treated nanoparticles
• 3 glass beaker 25 ml
• 3 laboratory plastic bottle caps
• 2 permanent rare earth magnets
• Aluminium lab clamps
• Laboratory stand
• Signal generator with variable voltage, current and frequency (min. req. 0–30 V, 0.1–2A, 1 Hz–50 kHz)
• Centrifuge (Eppendorf 5418 centrifuge, rotor No. FA-45-18-11)
• Eppendorf tubes
• 1 ml cuvettes for spectrophotometer
• VIS Spectrophotometer

### Procedure

1. In a glass beaker, in 30 ml of type 1 grade water, disperse MB treated NP.
 Aliquot suspension into 3 equal parts into 25 ml glass beakers.
2. Prepare a 2.5 cm high platform for a glass beaker by positioning three laboratory plastic bottle caps.
3. Between caps near the middle, place 2 permanent rare earth magnet stack 5 cm apart from one another. (Note: Stack is made from 5 pieces of 12 mm diameter button rare earth magnets.)
4. Immerse prepared needle in NP dispersion. Fix everything firmly in a position with aluminium lab clamps onto the laboratory stand.
5. Set current intensity at 100 mA and frequencies between 1 and 15 Hz. For each frequency use a different aliquot of NP suspension.
6. Collect 1 ml of suspension, pipette into 1 ml cuvettes and measure optical density (OD) at 580 nm using a spectrophotometer. (Note: NP are held in place with permanent magnets and no diffusion of NP into solution is to be expected).
7. In a glass beaker with NP suspension pipette same amount of Type 1 water to keep the volume constant.

Sample every 10 min for the first hour and once after two hours upon needle immersing. (Note: Same procedure can be utilized for different current settings. In the assessment of the methylene release behaviour, the cumulative amount of released MB was calculated, and the percentage of MB released was plotted vs. time).

## Cell cultivation for nanoparticles cytotoxicity testing in vitro

### Material

Note: Materials are listed in the order that they appear in procedure steps

• SH-SY5Y human neuroblastoma cell line (Sigma Aldrich, Saint Louis, Missouri, USA)
• Hera Cell 100 incubator (Thermo Fisher Scientific, Waltham, MA, SAD)
• Gas tanks with CO_2_
• DMEM/F12 1:1 with 15 mM HEPES, 2 mM L-glutamine and sodium bicarbonate (Sigma-Aldrich, Saint Louis, MO, SAD) CAT#D8437-500ML
• 100x non-essential amino acids (Sigma-Aldrich, Saint Louis, MO, SAD) CAT#M7145-100ML
• L-alanine-L-glutamine 200 mM (Sigma-Aldrich, Saint Louis, MO, SAD) CAT#G8541-100ML
• 100x Penicillin / Streptomycin solution (Sigma-Aldrich, Saint Louis, MO, SAD) CAT# P0781-100ML
• FBS (Sigma-Aldrich, Saint Louis, MO, SAD) CAT#F9665-500ML
• 15 ml conical tubes (Falcon^TM^, Corning, NY, SAD)
• Water bath
• Sterile Pasteur pipettes
• Centrifuge (Eppendorf 5418 centrifuge, rotor No. FA-45-18-11)
• Neubauer counting chamber
• Nunc™ EasYFlask™ Cell Culture Flasks, 25 cm^2^ growth area (Thermo Fisher, Waltham, Massachusetts, USA) CAT#156340
• Trypsin (Pan Biotech, Aidenbach, Germany) CAT#P10-029500Petry Dishes, 100 mm in diameter, pre-treated for cell adhesion (Greiner Bio-One, Kremsmünster, Austria) CAT#664 160
• 30% hydrogen peroxide
• Concentrated (96%) sulfuric acid
• Coverslips
• Two 300 ml wide throat Erlenmeyer flask made of Duran or Pyrex glass
• Nitrile glows
• Milli-Q^Ⓡ^ or other Type 1 ultrapure water
• Orbital shaker
• 100 ml of ACS or HPLC grade methanol
• Collagen type 1 from a rat tail (Upstate Biotechnology, Lake Placid NY, New York USA) CAT# 08-115
• 6 well plates (Sigma-Aldrich, St. Louis, MO, SAD)
• 24 well plates (Sigma-Aldrich, St. Louis, MO, SAD)
• 30% ethanol solution
• Laminar flow cabinet suitable for cell culture manipulation
• Incubator
• all-trans-retinoic acid (ATRA) (Fisher Scientific, Waltham, Massachusetts, USA)
• 10 mg/ml of iron nanoparticles
• 2 ml Eppendorf tubes
• Vortex (BioVorteks V1, Biosan, Riga, Latvia)
• Optical microscope capable of 100x total magnification

### Procedure

#### Cell cultivation

Cells were grown at 37 °C with 5% CO_2_ in a cell culture incubator.

To prepare a complete growth medium in 450 ml of DMEM/F12 following solutions are added:

▪ 1% of 100x non-essential amino acids
▪ 1% of 100x Penicillin / Streptomycin solution
▪ 0.5% of 200 mM solution of L-alanine-L-glutamine
▪ 50 ml of FBS. (Notes: Before adding, FBS is filtered through a sterile 0.2µm PES filter. This process removes any albumin polymers presented as cloudiness created by freezing, and we observed that cells grow better if FBS is filtered [9]; all solutions used for cell growth and manipulation are preheated to 37 °C to avoid a thermal shock)

1. 9 ml of growth medium is transferred to 15 ml Falcon tube.
2. Tube with frozen cells is defrosted by partially immersing it for 2 min in the water bath heated up to 37 °C.
3. The defrosted cell suspension is transferred by sterile Pasteur pipette into a growth medium in the Falcon tube.
4. Close the Falcon tube and holding it between thumb and index finger rotate gently twice to mix cells with growth medium.
5. The cell suspension is centrifuged at room temperature at 130 g for 5 min.
6. The supernatant is discarded, and cells are resuspended in 5 ml of fresh complete growth medium.
7. Cells are counted in the Neubauer chamber [10].
8. The final concentration of 100,000 cells/ml is prepared, and 7 ml of cell suspension is transferred to each 25 cm^2^ flask.
9. The next day growth medium is replaced with the same volume of a fresh one.
10. Cells are grown to 85% confluency.
11. After reaching the sufficiently confluent state, the medium is removed and cells are trypsinized with 2.5 ml of preheated trypsin solution for 3 min, gently shaking until cells are visibly detached from the bottom of the flask.
12. A fresh growth medium (2.5 ml) is added to trypsin/cell suspension to stop the enzyme and everything is transferred to a 15 ml falcon tube centrifuged at room temperature at 130 g for 5 min.
13. The supernatant is discarded, and cells are resuspended in 10 ml of fresh complete growth medium.
14. Cells are counted in the Neubauer chamber.
15. The final concentration of 250 000 cells/ml is prepared, and 12 ml of cell suspension is transferred to each 100 mm Petry dish.
16. Cells are grown to 85% confluency and then harvested for further experiments.

Notes: Since the neuroblastoma cells are adherent type, seeding concentration for experiments are calculated by growth surface area; we found that the optimal concentration is 50 000 cells per square centimetre. Areas per well are the following: 2 cm^2^ per well of 24 well plate; 9.6 cm^2^ per well of 6 well plates. Growth medium volumes per well are the following: 500 µL for each well of 24 well plates; 2000 µL for each well of 6 well plates. The final concentration of cells per ml the medium is the following: 200,000 cells/ml per well of 24 well plates, 240,000 cells/ml per well of 6 well plates.

#### Preparation of coverslips

1. Mix 1 part of 30% hydrogen peroxide with 9 parts of concentrated (96%) sulfuric acid. For 100 coverslips, 90 ml of sulfuric acid is to be mixed with 10 ml of hydrogen peroxide. The reaction is exothermic and releases elemental oxygen thus following precautions are to be made:
  ▪ use thermic shock-resistant glassware (Duran or Pyrex glass).
  ▪ perform mixing under a fume hood in a 300 ml wide throat Erlenmeyer flask made of Duran or Pyrex glass.
  ▪ always use nitrile glows, latex can be easily burned through by this solution.
2. Use gentle swirl motion to mix two solutions and wait for 10 min to settle.
3. In the second 300 ml wide throat Erlenmeyer flask place 100 coverslips (15 × 15 mm square, or 12 mm round) and pour into a mixture of sulfuric acid and hydrogen peroxide. Note that the choice of coverslip's size depends on the size of the well.

(Notes: Place the empty flask in a bucket (5 L) of distilled water to dilute residual acid and prevent unintentional chemical damage.)

4. Use gentle swirl motion to cover all coverslips with the solution. Repeat at least 3 times during the following 30 min. (Notes: During this period a significant amount of oxygen is created, manifested as bubbling. To prevent acid droplets sprinkling outside of the flask, it is advisable to cover it with a watch glass or with a glass made Petry-dish.)
5. After 30 min, pour acid (under the fume hood) from the flask in a bucket (5 L) of distilled water to safely dilute it and then pour 200 ml of clean distilled water in the flask with coverslips to dilute leftover acid.
6. Swirl the water in the flask with coverslips and then safely dispose of it to chemical waste, or safely dispose of it by diluting it with large amounts of tap water.
7. Pour in 200 ml of distilled water and place it on an orbital shaker (100 rpm) for 1 h.
8. After 1 h removes water from the flask and adds new 200 ml of distilled water. Repeat this washing cycle another 5 times – 6 h of washing in total.
9. After the last wash, pour out distilled water and dry the coverslips by adding 100 ml of ACS or HPLC grade methanol, swirl the flask for 5 min and then dispose of methanol in chemical waste.
10. Place aluminium foil over the flask opening and dry sterilize coverslips at 270 °C for 5 h.

#### Collagen type 1 growth surface coating

Collagen is applied in final concentrations of 5.5µg/cm^2^.

Different volumes of diluted collagen are used for different multiwell plates to speed up the drying: 250 µL for each well of 24 well plates; 750 µL for each well of 6 well plates.

1. Calculate the necessary volume of collagen stock solution by dividing the total mass of collagen (in micrograms) with the concentration of collagen stock solution μg/ml) – the given result is presented in millilitres.
  $$V\left(stock\;solution\right)=\frac{5.5\;\times \;\sum \begin{array}{c} well\;bottom\\ surface\;area \end{array}}{stock\;concentration}$$
2. Calculate the total volume of collagen working solution to be used for coating by multiplying the number of wells with the corresponding volume per well. - volumes per well are stated above
  V(workingsolution)=n(well)×V(specifficforwellsize)
3. Subtract the volume of collagen stock solution from the total volume of collagen working solution (given number is the volume of 30% ethanol).
  V(30%ethanol)=V(workingsolution)−V(stocksolution)
4. Mix collagen and ethanol on a vortex mixer.
5. Inside laminar flow cabinet pipette previously defined volume of diluted collagen solution in each well.
6. Once collagen is pipetted take the lids off, turn off laminar flow and close the cabinet then turn on the UV-C lamp for 1 h. After 1 h turn the lamp off.
7. Leave everything overnight in a laminar flow cabinet to completely dry.
8. The next day turns on the UV-C lamp in the laminar flow cabinet in 2 h duration to completely sterilize dried collagen and wells. (Note: Do not keep UV overnight because of UV-C mediated collagen degradation [11].)
9. Turn off UV-C lamp, turn on laminar flow and open the laminar flow cabinet.
10. After the proper aseptic procedure and by wearing disinfected gloves put the lids of multiwells back on and proceed with adding cell suspension.

Notes: Protocol is the same if coating coverslips for immunocytochemistry; before pipetting collagen solution, coverslips must be placed in each well with sterile tweezers; surface area remains unchanged since properly cleaned coverslips strongly adhere to the bottom of the well.

#### Cell differentiation and general experimental plan

▪ Day 0 – cells are seeded in collagenated wells and placed in an incubator.
▪ Day 1 – growth medium is replaced with fresh one containing 10 µM of All-trans retinoic acid (ATRA).
▪ Day 3 – growth medium is replaced with fresh one containing 10 µM of ATRA.
▪ Day 7 – growth medium is replaced with fresh one containing 10 µM of ATRA.
▪ Day 9 – growth medium is replaced with a fresh one with ATRA and without FBS.

**Fig. 1:**
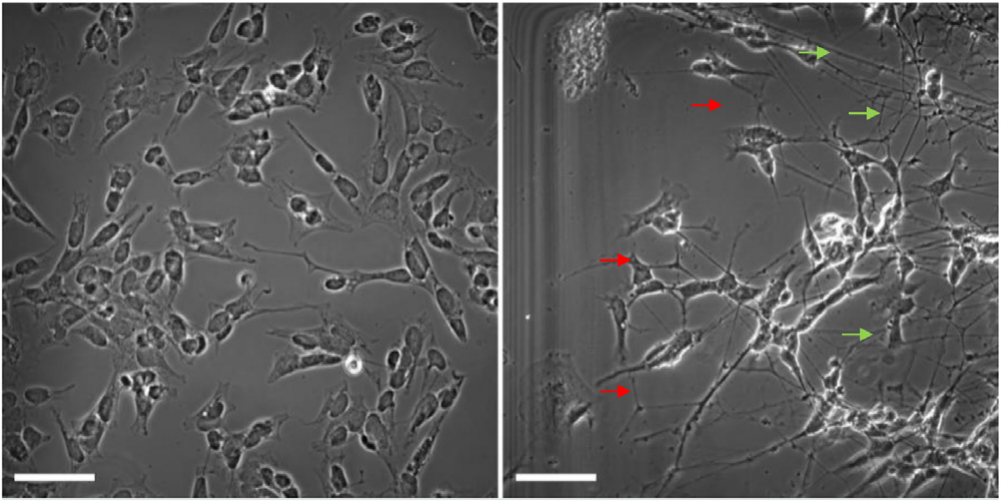
Comparison between undifferentiated and differentiated SH-SY5Y cells. The left image presents undifferentiated SH-SY5Y cells, the right image presents differentiated SH-SY5Y cells after 9-day treatment with 10 µM of retinoic acid. Note significant growth of neurites (red arrows) and formation of dense networks (green arrows) in differentiated cells and the absence of these morphological features in undifferentiated cells. Darkfield microscopy, Olympus fv1000 confocal microscope, 400x magnification; scale bar 50 µm.

Notes: Removal of FBS is necessary to remove the effect of cytokines in it. After differentiation cells change morphology. The presence of large neurite growth (Fig. 1.) is usually an indication of proper successful differentiation.

▪ Day 10 – growth medium is replaced with fresh one without FBS with added iron nanoparticles (see 5.2.5. chapter).
▪ Day 11 – end of the experiment (in the case of 24 h incubation) and go on with further experimental procedures.

#### Iron nanoparticle solution preparation

A stock solution of 10 mg/ml of iron nanoparticles (INP) is prepared in a cell growth medium without FBS.

1. Weight 10 mg of INP.
2. Place INP in sterile 2 ml Eppendorf tube.
3. Add 1 ml of growth medium.
4. Mix solution at the highest setting of vortex mixer for 30 s or until there are no clumps observed.
5. Calculate volumes of working solution for each concentration. (Notes: Take into consideration the volume of INP stock solution.)
6. Dilute INP stock solution in Falcon tubes of appropriate size.
7. Before preparing working solutions, iron nanoparticle stock solution must be mixed by repeated pipetting in and out 10 times.
8. Pipette working solutions of INP into each well. (Note: before pipetting into a well, INP solutions must be mixed by repeated pipetting in and out for 4 times).

## Gomori's Prussian blue reaction optimized for differentiated SH-SY5Y human neuroblastoma cell line

### Material

Note: Materials are listed in the order that they appear in procedure steps.

• Coverslips 15 × 15 mm (Carl Roth, Karlsruhe, Germany) Art. No. KHX0.1
• Coverslips 24 × 24 mm (Carl Roth, Karlsruhe, Germany) Art. No. KHX3.1
• Collagen type 1 from a rat tail (Upstate Biotechnology, Lake Placid NY, New York USA) CAT# 08-115
• Paraformaldehyde: buffered with PBS, pH adjusted to 7.40 (Acros organics, Fair Lawn, New Jersey, USA)
• PBS; 1x solution containing 137 mM of NaCl, 2.7 mM of KCl, 1.8 mM of KH_2_PO_4,_ 10 mM of Na_2_HPO_4;_ pH adjusted at 7.40
• Potassium ferrocyanide, p.a grade (Kemika, Zagreb, Croatia) CAS 13943-58-3
• Hydrochloric acid p.a grade; 36% (GramMol, Zagreb, Croatia) CAS 7647-01-0
• Filter paper
• Distilled water
• Nuclear fast red counterstain
• Ethanol absolute (Merck, Darmstadt, Germany) CAS: 64-17-5Sulphuric acid; CAS: 7664-93-9
• Resinous anhydrous mounting medium; Canada Balsam (Carl Roth, Karlsruhe, Germany) CAS 8007-47-4
• Microscope slides (Paul Marienfeld GmbH & Co.KG, Königshofen, Germany) CAT#1000200
• Zeiss AxioSkop 2Mot microscope with mounted Olympus DP 70 camera

### Procedure

**Fig. 2:**
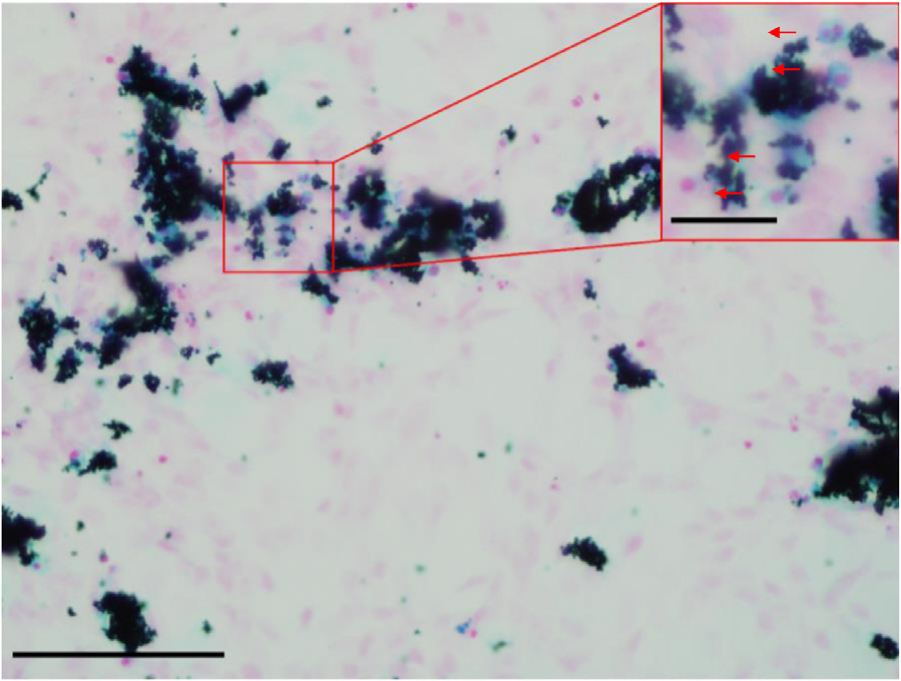
Prussian blue staining of differentiated SY-SY5Y cells 24 h post 0.1 mg/ml exposure to iron nanoparticles. Cell nuclei are stained pink to red and the positive Perls reaction is stained blue. Note blue-stained cytoplasm (red arrows) which contains an excessive amount of ferric (Fe^3+^) iron. Bright-field microscopy; Zeiss Axioskop 2MOT with mounted Olympus fluoview DP70 camera; 400x magnification; lower scale bar 100 µm, upper-scale bar 25 µm.

1. Wash coverslips and coat with collagen following the above-mentioned protocols.
2. Seed cells in previously mentioned number, differentiate for 9 days and treat the cells with INP (see 5.2.5. chapter).
3. Remove the growth medium.
4. Add 500 µL of 2 % buffered PFA.
5. Incubate at +4 °C for 30 min.
6. Remove PFA solution and wash 5 times with 1 × PBS.
7. Prepare 25 ml of fresh 2% aqueous solution of potassium ferrocyanide and mix with 25 ml of 4% hydrochloric acid.
8. Filter the solution through filter paper.
9. Remove PBS and pipette 500 µL of the prepared solution in wells and incubate at room temperature for 30 minutes.
10. Rinse 6 times in distilled water.
11. Stain with Nuclear fast red counterstain.
12. Remove the dye and rinse it until the water is clear.
13. Dehydrate in absolute ethanol, clear in xylene and mount on a microscope slide in resinous mounting media.
14. Cover with a coverslip. (Note: Positive reaction is presented as blue staining within cells (Fig. 2).)

## MTT viability assay optimized for differentiated SH-SY5Y human neuroblastoma cell line

### Material

Note: Materials are listed in the order that they appear in procedure steps.

• MTT powder (Santa Cruz Biotechnology, Dallas, Texas USA) CAT# sc-359848
• Falcon tube
• Tetrazolium bromide powder
• PBS; 1x solution containing 137 mM of NaCl, 2.7 mM of KCl, 1.8 mM of KH_2_PO_4,_ 10 mM of Na_2_HPO_4;_ pH adjusted at 7.40
• Ultrasonic homogenizer
• Ice bath
• 0.2 µm syringe PES filter
• 24 well plates (Sigma-Aldrich, St. Louis, MO, SAD)
• Incubator
• MTT solvent (Sigma-Aldrich, Saint Louis, MO, SAD) CAT# M-8910Collagen type 1 from a rat tail (Upstate Biotechnology, Lake Placid NY, New York USA) CAT# 08-115
• 2 ml Eppendorf tubes
• 1.5 ml Eppendorf tubes
• Centrifuge (Eppendrof 5418 centrifuge, rotor No. FA-45-18-11)
• Corning^Ⓡ^ 96 Well TC-Treated Microplates; flat bottom (Sigma-Aldrich, St. Louis, MO, SAD) CAT#CLS3595-50EA
• iMark microplate reader (Bio-Rad, Hercules, California, USA)

### Procedure

Nanoparticles are opaque and diffract light which presents a problem for any photometric reading. Since MTT assay is based on absorbance reading we had to modify the protocol [12,13]. Before viability test, an MTT stock solution must be prepared.

(Note: the stock solution is stable at least 6 months at -20 °C [14]).

#### MTT stock solution

1. Weigh 100 mg of tetrazolium bromide powder.
  (Note: this is enough for a total of 400 reactions performed in 24 well plates.)
2. Dissolve the powder in 20 ml of 1 × PBS.
  (Notes: to speed up the process using an ultrasonic homogenizer (70W of power, 15 s duration of the continuous pulse; falcon tube must be placed in an ice bath to keep solution cold).
3. Sterilize the solution by filtering it through a 0.2 µm syringe PES filter.
4. Make 1.5 ml aliquots and freeze at -20 °C.

#### MTT test procedure

1. Seed cells in collagenated 24 well plate using previously given instructions (see 5.2.3. chapter). (Note: use at least 6 biological replicas for each concentration and untreated control.)
2. Replace the growth medium with one containing INP and incubate for 24 h.
3. After 24 h pipette into each well 50 µL of MTT stock solution. (Note: this results in 0.5 mg/ml of tetrazolium bromide concentration per well.)
4. Gently mix growth medium and MTT stock solution in 24 well plates by a circular motion and place the plate in an incubator for 4-h to produce formazan crystals.
5. After incubation, in each well with MTT stock solution added, pipette 500 µL of MTT solvent and dissolve created formazan crystals by repeated pipetting in and out 10 times.
6. Transfer solution from each well into individual 1.5 ml Eppendorf tube and centrifuge at 15,000 g for 5 min at room temperature. (Note: this step is used to pellet all nanoparticles that may interfere with absorbance reading.)
7. After centrifugation, pipette from each Eppendorf tube 200 µL of supernatant into the individual well of flat bottom 96 well plate.
8. Read on a microplate reader at OD_595nm_.
9. Calculate OD percentage value against the untreated group.

Note: It is possible to use another well plate, like a 48 or 96 well plate to save the usage of the MTT stock solution. In that case, it is important to adjust the volume of the solutions. e.g., 200 μL solution is enough for the centrifugation, after centrifugation, pipette from each Eppendorf tube 100 μL of supernatant in 96 well plates.

## Antioxidant enzymes activities assay, and lipid peroxidation optimized for cell culture

### Materials

Note: Materials are listed so that they appear in procedure steps.

• 1.5 ml Eppendorf tubes
• Ice bloc or box with ice
• 100 mM phosphate buffer with 1 mM EDTA (pH 6.5)
• Vortex mixer
• Tissue grinder pestle specially designed to match microtubes
• 100 mM phosphate buffer (pH 7.0) containing 1 mM EDTA
• Tissue grinder pestle for 1.5 ml microcentrifuge tubes (Kimble, UNSPSC Code 41000000)
• Lambda 2 UV–Vis spectrophotometer equipped with a UV WinLab software package (Perkin Elmer, Wiesbaden, Germany).
• Spectrophotometer cuvettes, polystyrene (Merck)
• 0.036% H_2_O_2_ in 50 mM phosphate buffer (pH 7.0)
• 50 mM phosphate buffer (pH 7.0)
• 0.05 mM cytochrome C (Sigma-Aldrich, St. Louis, MO, SAD)
• 1 mM xanthine (Sigma-Aldrich, St. Louis, MO, SAD)
• Xanthine oxide (XOD, 0.1 U/mL) (Sigma-Aldrich, St. Louis, MO, SAD)
• 75 mM GSH (Sigma-Aldrich, St. Louis, MO, SAD)
• 30 mM CDNB (Sigma-Aldrich, St. Louis, MO, SAD)
• 100 mM phosphate buffer with 1 mM EDTA (pH 7.5)
• 2 mM GSSG (Sigma-Aldrich, St. Louis, MO, SAD)
• 2mM NADPH (Sigma-Aldrich, St. Louis, MO, SAD
• Ice-cold 1.15% KCl
• 8.1% sodium dodecyl sulphate
• 20% acetate buffer (pH 3.5)
• 0.8% thiobarbituric acid (TBA) (Sigma-Aldrich, St. Louis, MO, SAD)
• *n*-butanol-pyridine mixture (15:1, v/v)

### Procedure

#### Preparing cell homogenates

1. Transferee tubes with cells (see 5.2.1.) stored in a 1.5 ml Eppendorf tube to ice bloc or in the box filled with ice. (Note: Use at least 4 million cells per group/sample cells that are just collected from growth medium or previously-stored at -80 °C.)
2. Add 500 µL of ice-cold 100 mM phosphate buffer (pH 7.0) containing 1 mM EDTA in each Eppendorf tube containing cells.
3. Vortex each Eppendorf tube for 3–5 min.
4. Use tissue grinder pestle specially designed to match microtubes and homogenize cells with 20 up and down repeated motion. (Note: There should not be any pellet in the tube after homogenization. After this step cells could be also stored at -80 °C and analyzed later.)

#### Spectrophotometric analyses of antioxidant enzymes activities

The absorbance of all enzyme activity assays was recorded using a UV-Vis spectrophotometer.

Total soluble protein concentration in cell samples used for enzymes activities calculation was measured following the protocol described by Bradford (1976), using bovine serum albumin as a standard.

*Catalase (CAT; EC 1.11.1.6) activity*

1. Determine CAT activity using an H_2_O_2_ as a substrate [15].
2. Prepare reaction mixture (1.5 mL) directly in cuvette for spectrophotometry. Reaction mixture: Add 1450 µL of 0,036% H_2_O_2_ in 50 mM phosphate buffer (pH 7.0) and 50 µL of a sample/cell homogenat and vortex.
3. Measure the absorbance at 240 nm for 2 min.

Note: Absorbance will decrease due to H_2_O_2_ degradation. Prepare reaction mixture just before measurement.

4. Calculate CAT activity using the molar extinction coefficient (ε=0.04 mM/cm) and expressed as U/mg protein.

Activity = (ΔE X Vf) / (Δt x ɛ x Vs x d)
ΔE = change in absorbance; Vf = final volume of the reaction; Vs = volume of used enzyme extract; Δt = time of hydrolysis; ɛ = extinction coefficient; d = diameter of used cuvette.

*Superoxide dismutase* (SOD; EC 1.15.1.1) activity

1. Measure SOD activity as a degree of inhibition of cytochrome C reduction using superoxide radical [16].
2. Prepare reaction mixture (1.5 mL) directly in the cuvette for spectrophotometry just before measurement. Reaction mixture: 0.05 mM cytochrome C, 1 mM xanthine, 25 µL of XOD (0.1 U/mL) and 25 µL of a sample/cell homogenate.
3. Measure the absorbance at 550 nm during 3 min.
4. A calculated activity using the percentage of cytochrome C inhibition and express it as U/mg protein.

*Glutathione S-transferase* (GST; EC 2.5.1.13) activity

1. Estimate GST activity spectrophotometrically due to the formation of conjugates between 1-chloro-2,4-dinitrobenzene (CDNB) and reduced glutathione (GSH) [17].
2. Prepare reaction mixture (1.5 mL) directly in the cuvette for spectrophotometry just before measurement. Reaction mixture: 1450 µL of 100 mM phosphate buffer with 1 mM EDTA (pH 6.5), 50 µL of 75 mM GSH, 50 µL of 30 mM CDNB and 50 µL of a sample.
3. Measure the absorbance at 340 nm during 3 min.

Note: One-unit conjugates 1.0 μmole of 1-chloro-2,4-dinitrobenzene with reduced glutathione per minute at pH 6.5 and 25 °C. As conjugate formation occurred, there is an increase in absorbance.

4. Calculated GST activity using a molar extinction coefficient of the glutathione-1-chloro-2,4-dinitrobenzene conjugate (ε =9.6 mM/cm). The activity was expressed as U/g protein.

Activity = (ΔE X Vf) / (Δt x ɛ x Vs x d)
ΔE = change in absorbance; Vf = final volume of the reaction; Vs = volume of used enzyme extract; Δt = time of hydrolysis; ɛ = extinction coefficient; d = diameter of used cuvette.

*Glutathione reductase* (GR; EC 1.6.4.2) activity

1. Measure GR activity by the method described by Dolphin et al. [18] which is based on the reduction of oxidized glutathione (GSSG) in the presence of glutathione reductase (GR) and NADPH as a reducing agent.
2. Prepare reaction mixture (1 mL just before measurement) consisted of 400 µL of 100 mM phosphate buffer with 1 mM EDTA (pH 7.5), 500 µL of 2 mM GSSG, 50 µL of 2mM NADPH and 50 µL of a sample/cell homogenate.

Note: One unit reduces 1.0 μmol of oxidized glutathione per minute at pH 7.5 and 25 °C.

3. Measure the absorbance at 340 nm during 3 min.

Note: Due to NADPH consumption, the decrease in absorbance is monitored.

4. Calculated GR activity using the molar extinction coefficient for NADPH (ε =6.220 mM/cm). The activity was expressed as U/g protein.

Activity = (ΔE X Vf) / (Δt x ɛ x Vs x d)
ΔE = change in absorbance; Vf = final volume of the reaction; Vs = volume of used enzyme extract; Δt = time of hydrolysis; ɛ = extinction coefficient; d = diameter of used cuvette.

#### Determination of lipid peroxidation

1. LPO levels in collected cells were estimated by the method according to Ohkawa et al. [19] based on measuring thiobarbituric acid reactive substances (TBARS), especially malondialdehyde (MDA).
2. Homogenise cells in 500 µL ice-cold 1.15% KCl solution.

Note: Use the same homogenisation procedure described in the *Preparing cell homogenates* section.

3. Mix 150 µL of homogenate with 150 µL of 8.1% sodium dodecyl sulphate, 20% 125 µL of acetate buffer (pH 3.5) and with 0.8% 125 µL of thiobarbituric acid (TBA).
4. Incubate the mixture at 95 °C for 60 min. and cool for 15 min. at room temperature.

Note: During the heating of the acidic reaction mixture, the lipid peroxides decompose to form MDA that reacts with TBA. As a result, a red pigment will be formed.

5. Extract red pigment by adding 0.75 µL of water and 3.75 µL of the *n*-butanol-pyridine mixture (15:1, v/v).
6. Centrifuge the mixture at 4000 g for 15 min at 4 °C. Two layers will be separate.
7. Separate the red-coloured top layer and estimate its absorbance at 532 nm.
8. Express the results as nmol/g protein.

## Declaration of Competing Interest

The authors declare that they have no known competing financial interests or personal relationships that could have appeared to influence the work reported in this paper.
